# Knotless suture anchor fixation of Hahn–Steinthal fractures of the capitellum: A technical note with preliminary clinical outcomes

**DOI:** 10.1051/sicotj/2026035

**Published:** 2026-05-28

**Authors:** Michał Szufnara, Maciej Ciesielski, Błażej Wójtowicz, Jan Poszepczyński, Marcin Domżalski, Jędrzej Lesman

**Affiliations:** Department of Orthopaedics and Traumatology, University Clinical Hospital No. 2, Medical University of Łódź Łódź Poland

**Keywords:** Capitellum fracture, Hahn–Steinthal fracture, Osteochondral fracture, Knotless fixation, Elbow trauma

## Abstract

*Background:* Hahn–Steinthal fractures are rare osteochondral injuries of the humeral capitellum that primarily affect young and active patients. Although several fixation techniques have been described, most rely on metallic implants or knot-tying constructs that may increase intra-articular bulk and the risk of cartilage damage. The use of knotless suture anchor fixation for this specific fracture pattern has not yet been specifically described. To our knowledge, this study represents the first report describing knotless suture anchor fixation specifically for Hahn–Steinthal fractures of the capitellum. *Methods:* This study describes an open reduction and knotless suture anchor fixation technique for Hahn–Steinthal fractures of the capitellum and reports early clinical outcomes in a consecutive series of five patients treated in 2025. Functional outcomes were assessed using the Disabilities of the Arm, Shoulder, and Hand (DASH) score, while pain intensity was evaluated using the Visual Analog Scale (VAS). Elbow range of motion (ROM) and radiographic fracture healing were also assessed during follow-up. Patients were followed for 12 weeks postoperatively. *Results:* The cohort consisted of five patients (three men and two women) with a mean age of 37 years (SD 3.5). All fractures achieved radiographic union without secondary displacement. In addition, computed tomography performed at 3 months postoperatively confirmed osseous union and restoration of articular congruity in the index case. The mean DASH score improved from 62 preoperatively to 43 at 2 weeks, 31 at 6 weeks, and 21 at 12 weeks postoperatively. Mean VAS scores decreased from 8 preoperatively to 5 at 2 weeks, 4 at 6 weeks, and 3 at 12 weeks. The mean flexion–extension arc improved from 48° (SD 5.5) preoperatively to 110° (SD 6.7) at final follow-up. No intraoperative or postoperative complications were observed during the 12-week follow-up period. *Conclusions:* Open reduction followed by knotless suture anchor fixation represents a technically feasible technique associated with encouraging early clinical and radiological outcomes in this preliminary case series. The absence of intra-articular metal hardware may reduce the risk of cartilage damage while allowing stable fixation of small osteochondral fragments and early functional recovery.

## Background

Hahn–Steinthal fractures are rare osteochondral injuries of the humeral capitellum, characterized by a large displaced articular fragment and typically affecting young and active patients [1–3]. Capitellar fractures account for approximately 1% of all elbow fractures and represent a distinct subtype of coronal shear fractures of the distal humerus [2, 3]. Because of their intra-articular location and the limited fixation options available for osteochondral fragments, these injuries may present significant therapeutic challenges.

If inadequately treated, capitellar osteochondral fractures may lead to persistent pain, mechanical symptoms, joint incongruity, loss of motion, and early post-traumatic osteoarthritis [2, 3]. For this reason, anatomical reduction and stable fixation are generally recommended to restore the articular surface and allow early mobilization, thereby preserving elbow function [2].

Historically, open reduction and internal fixation with metallic implants has been considered the standard treatment for displaced capitellar fractures [2, 4]. However, screw- and pin-based fixation methods may be associated with implant prominence, iatrogenic cartilage injury, and in some cases the need for secondary implant removal procedures [5, 6].

Minimally invasive and arthroscopic techniques for capitellar fractures have also been described and may allow detailed assessment of the articular surface [5, 7–9]. Nevertheless, these procedures are technically demanding and may not be feasible in all clinical settings [7–9].

Various fixation strategies have been proposed for osteochondral fractures of the capitellum, including headless compression screws, Kirschner wires, and suture-based constructs [8–12]. While metallic implants provide rigid fixation, they may be less suitable for smaller osteochondral fragments and may increase the risk of intra-articular hardware-related complications [4, 13]. Suture-based fixation techniques offer a lower-profile alternative; however, many rely on knot-tying constructs that may still increase intra-articular bulk [11, 12].

Knotless suture anchor technology has gained increasing popularity in orthopedic surgery because it enables secure fixation while eliminating knot stacks and reducing implant prominence [14–16]. Favorable outcomes of knotless constructs have been reported in cartilage repair and ligament reconstruction procedures [17–21]. However, the use of knotless suture anchor fixation for Hahn–Steinthal fractures of the capitellum has not yet been specifically described [22–26].

Therefore, the aim of the present study was to describe an open reduction and knotless suture anchor fixation technique for Hahn–Steinthal fractures of the capitellum and to report the early clinical and radiographic outcomes in a consecutive preliminary case series.

## Methods

### Study design and patients

This retrospective case series included five consecutive patients treated for acute Hahn–Steinthal fractures of the capitellum between January and October 2025. All patients presented with a displaced osteochondral fragment of the capitellum considered suitable for surgical fixation. None of the patients had received prior conservative or surgical treatment of the affected elbow.

The study was conducted in accordance with the Declaration of Helsinki and was approved by the local Bioethical Commission. All patients provided written informed consent.

### Surgical technique

**Figure 1:**
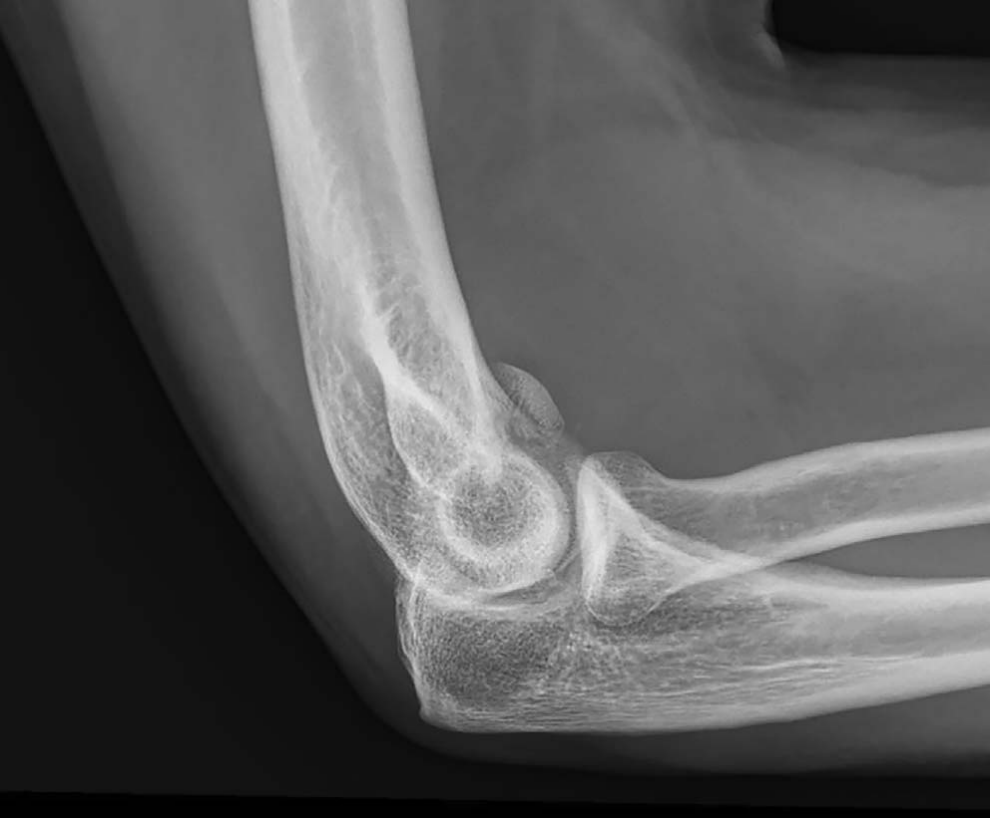
Preoperative anteroposterior and lateral radiographs demonstrating a displaced Hahn–Steinthal fracture of the capitellum with anterior displacement of the osteochondral fragment.

All procedures were performed under general anesthesia with the patient positioned supine. Preoperative anteroposterior and lateral radiographs confirmed a displaced Hahn–Steinthal fracture of the capitellum and were used for surgical planning (Figure 1). A standard lateral approach to the elbow was used to expose the capitellum of the distal humerus. Particular care was taken to identify and protect the posterior interosseous nerve during surgical exposure.

**Figure 2:**
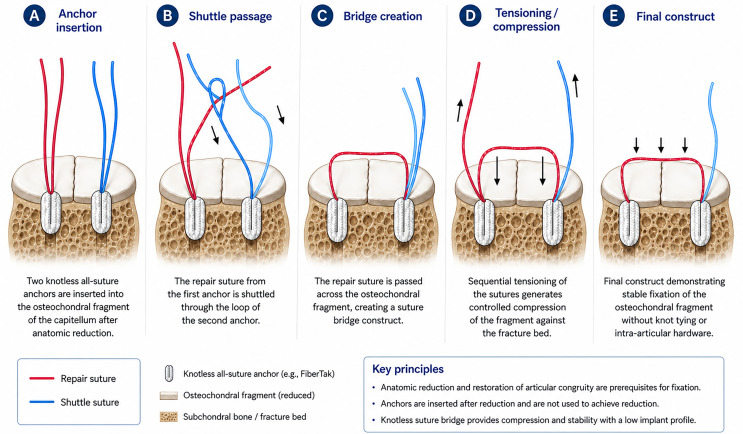
Schematic illustration of the reduction-first knotless fixation technique. (A) Insertion of two knotless all-suture anchors after anatomic reduction of the osteochondral fragment. (B) Shuttle passage of the repair sutures through the corresponding anchor loops. (C) Creation of the suture bridge construct across the fragment. (D) Sequential tensioning generates controlled compression across the fracture bed. (E) Final low-profile construct providing stable fixation without intra-articular metal hardware or knot stacks.

A schematic overview of the reduction-first knotless fixation technique is presented in Figure 2, illustrating the sequential steps of anchor insertion, suture shuttling, bridge formation, and final fragment compression.

After surgical exposure, the osteochondral fragment was identified and carefully mobilized. The fracture bed and the undersurface of the fragment were gently debrided to remove fibrous tissue and interposed debris while preserving viable subchondral bone and articular cartilage, thereby optimizing the biological environment for fracture healing.

**Figure 3:**
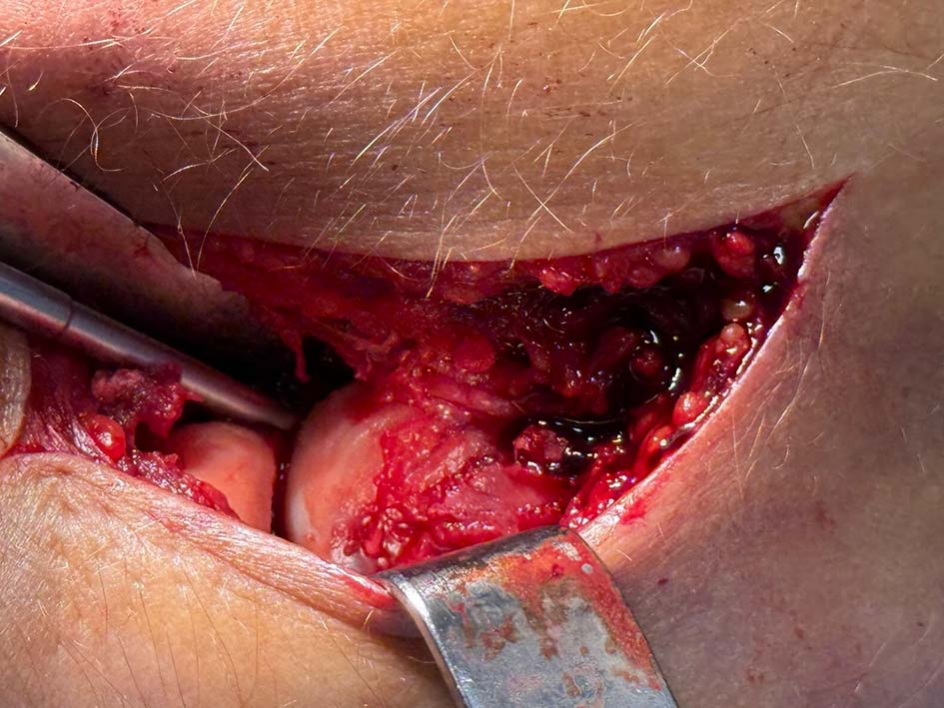
Intraoperative view demonstrating anatomic reduction of the osteochondral capitellar fragment under direct visualization prior to definitive fixation.

Anatomic reduction of the osteochondral fragment was achieved under direct visualization prior to any implant insertion. Restoration of the native articular contour and congruity of the capitellum was considered a prerequisite for definitive fixation. The fragment was temporarily stabilized using a probe, reduction clamp, or Kirschner wire, depending on fragment size and intraoperative stability, allowing precise assessment of reduction and fragment positioning (Figure 3).

**Figure 4:**
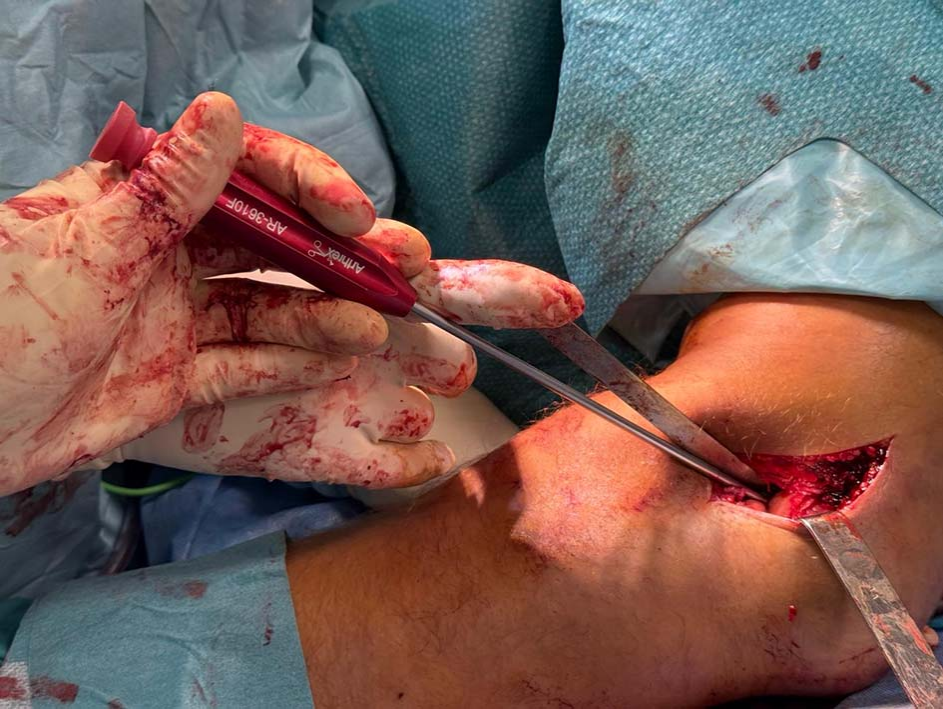
Insertion of two knotless FiberTak all-suture anchors into the subchondral bone of the reduced capitellar fragment, perpendicular to the fracture plane.

Only after satisfactory anatomic reduction had been confirmed were two knotless FiberTak all-suture anchors (Arthrex, Naples, FL, USA) inserted in a controlled manner into the subchondral bone of the capitellum, perpendicular to the fracture plane while avoiding penetration of the articular surface (Figure 4). This reduction-first strategy allowed accurate implant positioning relative to the fracture line and avoided the use of anchor tensioning as a reduction maneuver.

**Figure 5:**
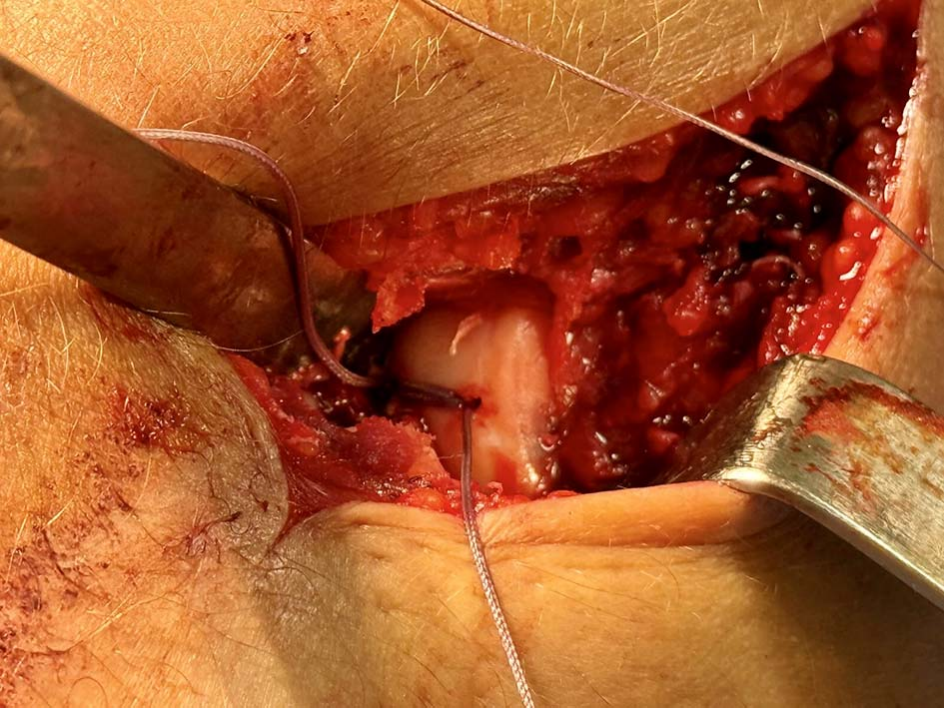
Final intraoperative view demonstrating restoration of the capitellar articular surface and stable knotless suture bridge fixation of the osteochondral fragment.

The repair sutures were subsequently shuttled through the corresponding anchor loops to create a low-profile knotless suture bridge construct across the osteochondral fragment. Sequential tensioning of the sutures generated controlled compression of the fragment against the fracture bed, providing stable fixation without knot tying or intra-articular metal hardware while maintaining anatomic reduction of the articular surface. The final construct was inspected intraoperatively, and fixation stability was assessed by direct manual testing and gentle passive elbow motion (Figure 5).

**Figure 6:**
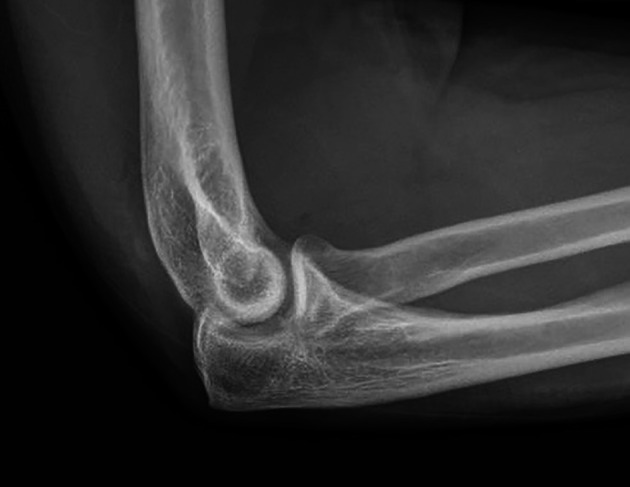
Postoperative anteroposterior and lateral radiographs confirming maintenance of anatomic reduction and stable fixation of the capitellar osteochondral fragment.

Final reduction and implant positioning were confirmed on postoperative radiographs (Figure 6).

### Postoperative management

Postoperatively, the elbow was immobilized in a posterior splint for a short period. Early passive and active-assisted range-of-motion exercises were initiated thereafter. Progressive strengthening exercises were introduced following radiographic evidence of fracture consolidation.

### Outcome assessment

Clinical outcomes were assessed using the Disabilities of the Arm, Shoulder, and Hand (DASH) score recorded preoperatively and at 2, 6, and 12 weeks postoperatively. Pain intensity was evaluated using the Visual Analog Scale (VAS) at the same follow-up intervals.

**Figure 7:**
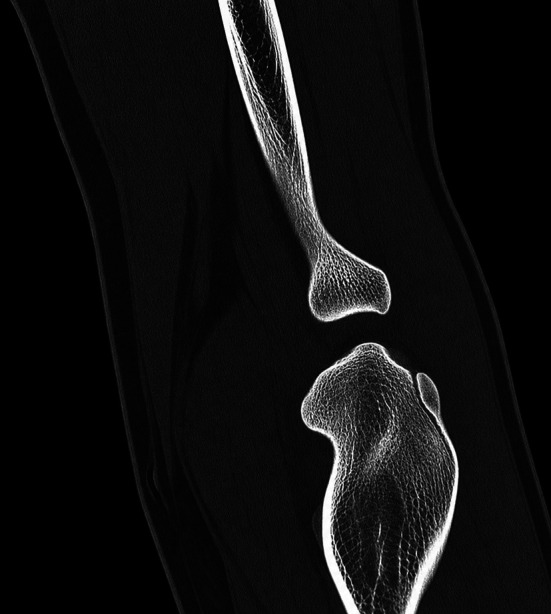
Three-month postoperative computed tomography demonstrating osseous consolidation of the capitellar fragment with restoration of articular congruity and maintained reduction.

Standard anteroposterior and lateral radiographs of the elbow were obtained during follow-up visits to assess fracture healing and implant position. Radiographic union was defined as the presence of trabecular continuity across the fracture site without secondary displacement of the osteochondral fragment. In the index case, computed tomography was additionally performed at 3 months postoperatively to confirm osseous union and restoration of articular congruity (Figure 7).

## Results

The study cohort consisted of five patients (three men and two women) with acute Hahn–Steinthal fractures of the capitellum. The mean patient age was 37 years (range, 32–42 years).

All fractures achieved radiographic union during the follow-up period without evidence of secondary displacement or fixation failure. In the index case, computed tomography performed at 3 months postoperatively confirmed osseous consolidation of the capitellar fragment with restoration of articular congruity (Figure 7).

No intraoperative or postoperative complications were observed, including implant-related irritation, neurovascular injury, or loss of fixation. No cases of fragment resorption or secondary displacement were identified during follow-up.

Clinical outcomes demonstrated progressive improvement over time. The mean DASH score improved from 62 preoperatively to 43 at 2 weeks, 31 at 6 weeks, and 21 at 12 weeks postoperatively. Similarly, the mean VAS score decreased from 8 preoperatively to 5 at 2 weeks, 4 at 6 weeks, and 3 at 12 weeks, reflecting gradual pain reduction and functional recovery.

The elbow range of motion improved substantially following surgical treatment. The mean flexion–extension arc increased from 48° preoperatively (SD 5.5) to 110° (SD 6.7) at final follow-up.

Radiographic evaluation confirmed maintenance of anatomic reduction and stable fixation in all patients throughout the follow-up period.

## Discussion

The principal finding of this study is that open reduction followed by knotless all-suture anchor fixation of Hahn–Steinthal fractures of the capitellum is technically feasible and associated with encouraging early clinical and radiological outcomes. All patients achieved fracture union, demonstrated progressive functional improvement, and experienced gradual pain reduction during follow-up. In the index case, postoperative computed tomography at 3 months confirmed osseous consolidation of the osteochondral fragment with restoration of articular congruity, supporting the biological feasibility of fragment healing with this fixation strategy.

Capitellar fractures remain challenging intra-articular injuries because successful treatment requires precise restoration of the articular surface while maintaining stable fixation of relatively small osteochondral fragments [4, 21]. Anatomical reduction is essential regardless of the fixation method used, as restoration of joint congruity directly influences elbow kinematics and long-term joint preservation [2, 5]. Traditional fixation techniques include headless compression screws, Kirschner wires, and bioabsorbable implants [5, 13]. Among these, headless compression screws remain the most commonly reported technique and are generally considered the standard fixation method because they provide rigid interfragmentary compression and allow early mobilization [5, 23].

Clinical outcomes after headless screw fixation have generally been favorable. Dubberley et al. reported satisfactory functional outcomes after open reduction and internal fixation of capitellar fractures, while Ruchelsman et al. demonstrated reliable union and restoration of elbow function using buried headless screws [2, 5]. More recently, Tanrıverdi et al. confirmed favorable outcomes with headless screw fixation, further supporting its role as an established treatment option [23]. However, despite these advantages, screw fixation may be limited in smaller osteochondral fragments, where implant insertion may increase the risk of fragment fragmentation, hardware prominence, or articular cartilage violation [4, 5, 13].

Fragment preservation is an important principle in the treatment of reconstructable osteochondral capitellar fractures. Preservation of the native osteochondral fragment allows restoration of the articular surface and maintenance of joint congruity, which may be preferable to fragment excision whenever stable fixation can be achieved [24, 25]. This concept is supported by the broader osteochondral repair literature, in which preservation and fixation of viable fragments have been associated with improved joint biomechanics and better long-term cartilage preservation [25].

In this context, the knotless fixation strategy described in the present study offers several potential advantages. Unlike screw-based fixation, the use of all-suture anchors eliminates rigid intra-articular metal hardware and may therefore reduce the risk of iatrogenic cartilage injury. Suture-based fixation techniques have previously been described for capitellar and radial head osteochondral injuries with encouraging clinical outcomes [11, 12]. Runer et al. demonstrated the feasibility of transosseous all-suture fixation for osteochondral flake refixation, while Li et al. reported satisfactory outcomes using suture anchor fixation in capitellar osteochondral fractures [11, 12].

A further advantage of the present technique is the use of a knotless suture bridge configuration. This construct enables controlled fragment compression while eliminating intra-articular knot stacks, thereby minimizing implant prominence and reducing the risk of soft-tissue irritation. Low-profile knotless fixation strategies have shown promising results in cartilage repair and osteochondral fixation procedures in other joints [14–16, 26]. Biomechanically, suture bridge constructs may improve compression distribution across the osteochondral fragment and enhance fixation stability while preserving fragment integrity [26, 27].

A key technical aspect of the present method is the reduction-first strategy. In contrast to fixation methods in which reduction is achieved through implant tensioning, the fragment in our technique was anatomically reduced prior to anchor insertion. This sequence allows direct visual confirmation of articular congruity and more precise implant placement relative to the fracture line. In our opinion, this technical sequence may reduce the risk of malreduction and improve reproducibility, particularly in smaller or more fragile osteochondral fragments.

The present study has several limitations. First, the retrospective design and small sample size limit the generalizability of the findings and preclude comparative analysis with established fixation methods. Second, although radiographic union was observed in all patients and computed tomography confirmed osseous healing in the index case, advanced imaging was not systematically performed in the entire cohort, limiting comprehensive assessment of fragment viability and cartilage integrity. Third, the follow-up period remains relatively short and does not allow assessment of long-term complications such as post-traumatic osteoarthritis, avascular necrosis, or late fragment resorption. Finally, the absence of a control group treated with conventional fixation methods prevents direct comparison with established techniques. Nevertheless, given the rarity of Hahn–Steinthal fractures, preliminary technical series remain valuable for the development of alternative fixation strategies and may help guide future comparative studies [21, 22].

## Conclusions

Open reduction followed by knotless all-suture anchor fixation represents a technically feasible and reproducible technique for the treatment of Hahn–Steinthal fractures of the capitellum. This approach allows stable fixation of osteochondral fragments while avoiding intra-articular metal hardware, potentially reducing implant prominence and the risk of iatrogenic cartilage injury.

The technique may be particularly useful in smaller osteochondral fragments that are not ideally suited for screw fixation because of fragment size or risk of fragmentation. In this preliminary case series, knotless fixation provided stable fragment fixation and was associated with encouraging early clinical and radiological outcomes, including progressive functional recovery and radiographic fracture union.

Further studies involving larger patient cohorts, systematic advanced imaging assessment, and longer follow-up are required to evaluate long-term joint preservation and to compare this technique with established fixation methods.

## Data Availability

The datasets generated and analyzed during the current study are available from the corresponding author on reasonable request.
